# Regional Soil and Soybean-Associated Bacterial Communities Are Linked to Larval Nutritional Composition in the Edible Insect *Clanis bilineata tsingtauica*

**DOI:** 10.3390/insects17070746

**Published:** 2026-07-21

**Authors:** Zong-Nan Li, Qing-Yi Li, Qiu-Lin Wu, Su-Fen Cui, Pan Deng, Ji-Hu Li, Jian-Jun Guo, Lei Qian, Huai-Jian Liao

**Affiliations:** 1 Institute of Leisure Agriculture, Jiangsu Academy of Agricultural Sciences, Nanjing 210014, China; 2 College of Biotechnology, Jiangsu University of Science and Technology, Zhenjiang 212100, China; 3 Jiangsu Provincial University Key Laboratory of Agricultural and Ecological Meteorology, School of Ecology and Applied Meteorology, Nanjing University of Information Science and Technology, Nanjing 210044, China; 4 School of Grain Science and Technology, Jiangsu University of Science and Technology, Zhenjiang 212004, China; 5 Institute of Nanfan and Seed Industry, Guangdong Academy of Sciences, Guangzhou 510000, China; 6 Institute of Entomology, Guizhou University, Guiyang 550025, China

**Keywords:** *Clanis bilineata tsingtauica*, regional soil background, soil nutrients, soybean bacteria, gut bacteria, larval nutritional composition, amino acids, fatty acids

## Abstract

Regional soil conditions may affect edible insect nutrients through associated changes in host plants and bacterial communities, but these relationships remain poorly understood. Here, we compared soils, soybean leaves, larval gut bacteria, and nutritional composition of *Clanis bilineata tsingtauica* across three sites. Soil nutrient parameters were measured before and after larval inoculation, bacterial communities were analyzed in soil, leaves, and larvae, and larval amino acid, protein, and fatty acid profiles were determined. Available potassium increased 1.17-fold in Lianyungang; several nutrients decreased in Huaian, whereas soil pH decreased by 4.0% in Nanjing. Larvae from Lianyungang contained higher levels of several amino acids (Serine, Glycine, etc.), whereas those from Huaian had higher saturated and unsaturated fatty acid contents (Stearic acid, Elaidic acid, etc.). Genus-level overlap analysis showed partial taxonomic overlap among soil, leaves, and larvae, and the number of shared genera generally declined from soil to leaves to larvae, consistent with compartment-specific filtering. Correlation analyses further indicated that soil nutrients, especially available phosphorus and available potassium, were significantly correlated with dominant bacterial genera and with specific larval amino acid and fatty acid traits. Overall, these findings suggest that regional soil background is associated with coordinated variation in soybean-associated bacteria, larval gut bacteria, and the nutritional composition of *C. bilineata tsingtauica* larvae.

## Simple Summary

The nutritional composition of edible insects can vary substantially by location, although the underlying causes are often unclear. Here, we investigated how soil background conditions and associated bacteria are related to variation in the nutritional composition of the edible soybean hawkmoth across three study sites of eastern China. After larval introduction, soil properties responded differently among sites: one exhibited higher level of pant-available potassium, another showed a broad decline in nutrient levels, and the third showed lower soil pH. Consistent with these soil differences, larval nutritional composition also varied by site. Larvae from one site contained higher concentrations of several amino acids, whereas larvae from another site showed higher fatty acid contents. We further observed pronounced differences among the sites in bacterial communities across soil, soybean leaves, and larval midgut, with the number of shared bacteria generally decreasing from soil to leaves to larvae. Moreover, soil nutrients were associated with both larval nutrient profiles and dominant bacterial genera. Overall, these findings indicate that the nutritional composition of this edible insect is linked not only with its host plant, but also with soil background conditions at the three study sites and bacterial communities. This knowledge could improve edible insect production, enable more stable food quality, and support sustainable insect farming.

## 1. Introduction

Microorganisms are ubiquitous across plant tissues, animal hosts, and terrestrial and aquatic ecosystems. The composition and diversity of microbial communities not only reflect the conditions of their habitats but may also influence local ecological processes through interactions with hosts, soil properties, and other microorganisms [1]. Bacterial component assemblages play key roles in shaping soil microenvironments, mediating metabolism, enhancing plant stress resistance, and regulating insect metabolism [2,3]. Accumulating evidence shows that some bacteria can be translocated from soil to plant roots, stems, and leaves, with their abundance typically decreasing along this pathway. After selective colonization of plant tissues, these bacteria may be ingested by insects and subsequently detected in the gut, forming a potential soil–plant–insect transmission route [4]. Within this framework, soil-derived bacteria promote plant growth and defense; plant-associated bacteria enhance nutrient acquisition and stress tolerance, and insect gut bacteria improve host immunity and metabolic regulation [5,6,7].

Although existing studies have clarified how soil-derived bacteria help insects overcome plant defenses and insecticides, their roles in insect nutritional metabolism remain insufficiently understood. Insect nutritional metabolism is co-regulated by multiple factors, including the host plant, the gut bacteria, and environmental conditions. In agricultural production, substantial differences in physiological indicators are often observed among geographical populations of the same insect species, and these differences are closely linked to the regional environment and to the nutritional status of local host plants [8]. Differences in soil texture, parent material, pH, organic matter content, and nutrient composition are correlated with distinct soil bacterial communities across regions [9]. Collectively, soil nutrients and resident bacteria can directly affect the nutritional composition of host plants and the accumulation of plant secondary metabolites [10,11]. For example, Wang found that variations in the cultivation areas of daylily resulted in significant differences in plant protein, amino acid, and other metabolite contents; importantly, the regional factors had a greater impact on the plant bacterial community than did differences among plant parts [12,13]. However, it remains unclear whether these soil-driven plant differences are transmitted to the next trophic level via insect feeding, thereby influencing insect growth and development, nutritional metabolism, and gut bacteria. Recent evidence suggests that bacterial transmission is not confined to the soil–plant–insect pathway. Symbiotic bacteria found in insects typically originate from the soil, and insects can directly acquire these functionally specific bacteria from the soil environment. Although these bacteria may not directly control growth and development, they help hosts detoxify dietary toxins and antinutritional factors and provide essential nutrients that the host cannot synthesize de novo, thereby supporting host survival [14,15,16]. For example, *Acinetobacter* AS23, a gut symbiont that confers tea saponin tolerance to *Curculio chinensis*, is predominantly soil-derived: female weevils acquire it during pupation and transmit it vertically to their offspring [17]. Similarly, *Riptortus clavatus* acquires *Burkholderia* from the soil upon adulthood, increasing body weight and several morphological traits, including body length and thorax and abdomen width [18]. *R. clavatus* also uses *Burkholderia*’s fenitrothion-degrading enzymes to metabolize organophosphorus insecticides [19].

Certain edible insects, such as mealworm larvae, contain approximately 24% protein—comparable to lean beef—and exhibit a digestibility rate exceeding 75% [20]. These characteristics make them a highly promising alternative protein source for both the feed and food industries. Actually, edible insects a long history of consumption across Asia, Africa, and Latin America [21,22]. Among them, the soybean hawkmoth (*Clanis bilineata tsingtauica*), a Lepidopteran species in the family *Sphingidae*, has developed into a rapidly growing industry in China, with the market size has exceed one billion yuan by 2025 [23]. Geographically, the *C. bilineata tsingtauica* industry is concentrated in Jiangsu, Henan, Shandong, and Anhui provinces and is gradually expanding into southern regions, including Hainan, Guangdong, and Yunnan [24]. In Jiangsu, the industry’s birthplace, Lianyungang serves as the core trading and consumption hub, Huaian has emerged as breeding base in recent years, and Nanjing functions as a key center integrating farming practices and academic research [25]. In agricultural production, *C. bilineata tsingtauica* larvae are oligophagous, parasitizing legumes (e.g., soybean and *Pueraria* leaves), black locust, and vine plants [26]. Previous studies have shown that host plant species significantly affect larval feeding preference and nutritional status: Qian et al. (2023a) identified the soybean variety “Ruidou No. 1” as the optimal host plant for *C. bilineata tsingtauica* larvae [27]. Consistently, Xie (2023) reported that larvae fed *Pueraria* leaves had higher crude protein and crude fat contents, a greater proportion of essential amino acids, and higher gut bacteria diversity than larvae fed soybean leaves [26]. Together, these findings indicate that host plants, as direct food sources, are linked to the nutritional composition of *C. bilineata tsingtauica* larvae. Moreover, our previous work demonstrated that *Enterococcus casseliflavus*, a midgut symbiont of *C. bilineata tsingtauica* larvae, regulates the host’s arginine metabolic pathway [28,29]. Building on these advances, it is evident that within the *C. bilineata tsingtauica* industry, complex interactions among soil properties, soybean nutrition and defense, larval physiology, and associated bacteria collectively influence larval growth, development, and nutritional value. Accordingly, two key questions remain: do soil physicochemical properties in different sites influence the endophytic bacteria in soybean by shaping the soil bacterial community? And do these soybean-derived differences subsequently influence larval nutritional composition and gut bacteria?

The aim of this study was to examine the relationship between soil background conditions, associated bacterial communities, and variation in the nutritional composition of *C. bilineata tsingtauica* larvae across three study sites in eastern China. Specifically, we compared soil physicochemical properties, bacterial communities in soil, soybean leaves, and larval guts, as well as larval nutritional traits, and evaluated the associations among these variables. By adopting this approach, the study offers an integrated perspective on the soil–soybean–larva system and highlights multitrophic associations that may affect larval nutritional composition. This work extends our previous findings on *E. casseliflavus*-mediated regulation of arginine metabolism. It also provides a broader view of the multitrophic interactions potentially linked to variation in larval nutritional composition.

## 2. Materials and Methods

### 2.1. Insect Stocks

In this study, eggs of the soybean hawkmoth *C. bilineata tsingtauica* (populations with stable heredity for more than three generations) were obtained from Yuntai Farm in Jiangsu Province (Lianyungang, Jiangsu, China; 119.29° E, 34.59° N). The insects had been reared indoors for multiple generations to ensure consistency of the larval population source. *C. bilineata tsingtauica* larvae were reared on soybean plants grown under a 27 °C/16 h light and 25 °C/8 h dark photoperiod, with a relative humidity (RH) of 75%. Soybean (*Glycine max* L.) seeds were supplied by the Legume Crops Laboratory (Nanjing, China).

### 2.2. Experimental Design and Sample Collection

To investigate variation in soil physicochemical properties, bacterial communities linked to soil, soybean leaves, and larval guts, as well as larval nutritional composition under field conditions, three geographically distinct soybean-growing locations in Jiangsu Province were selected for rearing experiments: Lianyungang (LYG, 119.24° E, 34.27° N), Huaian (HA, 119.27° E, 33.78° N), and Nanjing (NJ, 118.47° E, 32.06° N). These sites were chosen due to their differing significance: LYG is the native habitat of *C. bilineata tsingtauica* and serves as the main market for the related industry; HA has recently emerged as a new production base; and NJ, located in southern Jiangsu, is warmer than the other two regions and was also used for laboratory validation experiments. Regarding soil classification, NJ was characterized as yellow-brown soil, HA as fluvo-aquic soil, and LYG as cinnamon soil with local salinization. One field site was investigated in each region. Within each site, sampling and larval inoculation were conducted in a uniform soybean field plot under natural, in situ conditions. Thus, the three locations in this study represent three geographically separated field sites rather than replicated regional blocks. The study was designed to compare site-associated patterns across representative production locations, but it does not enable the statistical separation of regional effects from site-specific factors such as agronomic history, microclimate, soil legacy, or initial bacterial composition.

Soybeans were sown directly in the field on 15 March 2024, rather than in containers or under controlled-environment conditions. The same soybean seed source was used across locations. During the trial period, all plots received broadly comparable basic management. During the growing season, minimum temperatures ranged from 8 to 14 °C, and maximum temperatures from 12 to 26 °C. Wind speeds were typically between 1 and 3 on the Beaufort scale, and the average daily sunshine duration was 10–12 h. Compound fertilizer (elements N:P:K = 3:8:4) was applied at 150 kg/ha. Additionally, 40-mesh insect netting was installed 2.5 m above the fields to control natural enemies. No artificial irrigation was applied, and soil moisture depended on natural precipitation. However, because the experiments were conducted under open-field conditions in different locations, local background conditions and farming history could not be completely standardized or eliminated. Likewise, the study did not adopt a randomized block design across multiple independent sites within each region.

The number of larvae surviving to the fifth instar was not systematically recorded in this experiment. Based on routine rearing experience under comparable field conditions, approximately 50% of individuals typically reach the fifth instar; however, this value was not quantified as an experimental endpoint in the present study. On the 35th day after sowing, control samples were collected before larval inoculation for subsequent comparisons. Six intact soybean plants were independently sampled for leaf and rhizosphere soil collection from each experimental field site. For each plant, both leaf and rhizosphere soil samples were obtained and treated as individual biological replicates. To minimize sampling bias, soil samples from all sites were collected using a standardized protocol at consistent soil depths and were processed under identical analytical conditions. Control measurements obtained prior to inoculation served as site-specific references for evaluating post-inoculation changes. Leaf samples were placed on ice during transport to the laboratory. To specifically characterize the leaf endophytic bacteriome and minimize interference from epiphytic bacteria, leaf surfaces were sterilized prior to DNA extraction. Briefly, leaves were rinsed with sterile water for 0.5 min, washed with 75% ethanol for 1 min, treated with 2% NaClO for 3 min, rinsed again with 75% sterile ethanol for 1 min, then finally rinsed with sterile water for 0.5 min before being stored at −80 °C. Whole plants were then collected, with bulk soil removed from the roots, and plants transported on ice. Loosely adhering soil was discarded, and soil tightly associated with roots was collected using a sterile brush. For soil samples, soil from the roots of six plants was collected, flash-frozen in liquid nitrogen, and stored at −80 °C. For each biological replicate of plant/soil samples, the sample consisted of a mixture of three out of six plant/soil samples. After control sampling, the remaining soybean plants in the same field plot were used for *C. bilineata tsingtauica* larval rearing. Each soybean plant was inoculated with 50 eggs, and the larvae were allowed to develop for 25 days until they reached the mature fifth instar stage. The inoculation density of 50 eggs per plant was selected to reflect current field-rearing practices in this system; however, this density may also influence soybean physiology, endophytic bacterial community, and food quality. Because larvae could move among neighboring soybean plants after hatching, individual fifth-instar larvae could not be reliably assigned to a single plant of origin. Larvae used for pooling were healthy individuals randomly collected from the inoculated plot at each site rather than being tracked to individual source plants. Whole larvae and dissected midgut tissues were collected separately. Pooled biological replicates were drawn from the same inoculated field plot within each site. For each biological replicate, two whole larvae were pooled together and stored at −80 °C. Another leaf and rhizosphere soil samples were collected again from the inoculated plot using the same protocol described above.

Importantly, the pre- and post-inoculation comparisons in this study represent two sampling time points from the same field plot within each site, rather than repeated measurements from the same individual plants. The “control” samples referred to in the revised manuscript are therefore baseline, pre-inoculation samples, not synchronous end-point no-larva controls. No separate larva-free plot was maintained and sampled at the final time point. As a result, temporal changes observed between pre- and post-inoculation samples may reflect not only larval activity, but also plant development, seasonal progression, and natural rhizosphere dynamics. For bacteriome analysis and soil physicochemical property assessment, plants and rhizosphere soil samples collected before and after inoculation with *C. bilineata tsingtauica* larvae were used as control and treatment groups, respectively, enabling a clearer evaluation of how larval feeding influences soybean endophytic bacteria and root-associated soil communities. Bacteriome analysis was performed on samples from soil, leaves, and the guts of *C. bilineata tsingtauica* larvae, with six biological replicates per group. For the analysis of soil physicochemical properties, five replicates per group were prepared, with 5 g of soil used for each replicate. Larval nutritional composition was also assessed with five replicates per group. Because samples collected before and after inoculation were obtained from different plants within the same plot and were not individually paired, statistical analyses were conducted as comparisons between independent replicate groups at each sampling stage, rather than as strictly paired observations. All measurements were statistically compared using the same analytical framework.

### 2.3. Larval Dissection and Midgut Sample Preparation

Mature fifth-instar larvae of *C. bilineata tsingtauica* were randomly selected for dissection. Whole larvae and dissected midgut samples were processed separately to facilitate distinct downstream analyses. Whole larvae were utilized for nutritional composition analysis, while dissected midgut samples were employed for gut bacteriome profiling. Prior to dissection, the larvae were starved for 12 h to empty their guts. Larvae were surface sterilized by immersion in 75% ethanol for 1 min and then rinsed thoroughly with sterile water to remove visible surface impurities remained. Dissections were carried out under aseptic conditions using sterilized scissors and forceps. The midgut was carefully removed, transferred into sterile 2 mL cryotubes, flash-frozen in liquid nitrogen, and stored at −80 °C for subsequent analysis of bacterial community composition. Each gut bacteriome biological replicate consisted of three pooled midguts.

### 2.4. Bacteriome Community Composition

The bacterial community composition in soil soybean leaf, and larval gut samples was characterized using 16S rRNA gene amplicon sequencing. For larval bacteriome analysis, DNA was extracted from dissected midgut samples rather than from whole larvae or external body surfaces to ensure accurate profiling of gut-associated bacteria. Total bacterial genomic DNA was extracted from soil, larval midgut and soybean leaf samples using the E.Z.N.A.^®^ Soil DNA Kit (Omega Bio-tek, Norcross, GA, USA) according to the manufacturer’s instructions. DNA quality and concentration were assessed by 1.0% agarose gel electrophoresis and NanoDrop 2000 (Thermo Scientific, Waltham, MA, USA). To reduce the amplification of host-derived DNA and enhance detection of bacteria, different PCR strategies and 16S rRNA hypervariable regions were employed across sample types. For soil and larval gut samples, the V3-V4 hypervariable region of the bacterial 16S rRNA gene was amplified, which is widely used for bacterial community profiling in samples with minimal host DNA interference. The primers 338F (5′-ACTCCTACGGGAGGCAGCA-3′) and 806R (5′-GGACTACHVGGGTWTCTAAT-3′) were used with the T100 Thermal Cycler (Bio-Rad, Hercules, CA, USA). PCR products were purified and recovered by 2% agarose gel electrophoresis, then quantified using a Qubit 4.0 fluorometer (Thermo Fisher Scientific, Waltham, MA, USA).

For the soybean leaf endophytic bacteriome, a nested PCR strategy targeting the V5–V7 hypervariable region of the 16S rDNA gene was implemented to minimize co-amplification of plant chloroplast DNA and thus improve detection of bacterial sequences in host-rich tissues. The first round of PCR used primers 799F (5′-AACMGGATTAGATACCCKG-3′) and 1392R (5′-ACGGGCGGTGTGTRC-3′), followed by a second round with primers 799F (5′-AACMGGATTAGATACCCKG-3′) and 1193R (5′-ACGTCATCCCCACCTTCC-3′). PCR products were recovered and purified using 2% agarose gel electrophoresis and quantified using a Qubit 4.0 fluorometer. PCR was performed in 20 μL reactions containing 4 μL 5× FastPfu buffer, 2 μL 2.5 mM dNTPs, 0.8 μL each primer (5 μM), 0.4 μL FastPfu polymerase, 10 ng template DNA, and nuclease-free water. The cycling program consisted of 95 °C for 3 min; 27 cycles of 95 °C for 30 s, 55 °C for 30 s, and 72 °C for 45 s; followed by 72 °C for 10 min. Purified amplicons were pooled in equimolar amounts and sequenced on an Illumina NextSeq 2000 platform (Majorbio Bio-Pharm Technology Co., Ltd., Shanghai, China).

Raw FASTQ files were demultiplexed and quality-filtered using fastp v0.19.6 and merged using FLASH v1.2.7. Reads were truncated at sites with an average quality score < 20 in a 10 bp sliding window, and truncated reads shorter than 50 bp or containing ambiguous bases were discarded. Only reads with overlap lengths > 10 bp were assembled, and reads failing assembly were removed. Optimized sequences were clustered into OTUs at 97% sequence similarity using UPARSE 11.0.667, and the most abundant sequence in each OTU was selected as the representative sequence. Taxonomic annotation was performed using RDP Classifier v2.2 against the SILVA 16S rRNA database (v138.2) with a confidence threshold of 0.7. Chloroplast sequences were removed from the OTU table prior to downstream analyses. To reduce the effect of unequal sequencing depth, all samples were rarefied to 20,000 sequences per sample, which retained an average Good’s coverage of 99.09%. Alpha-diversity indices were calculated using Mothur v1.30.1, and beta-diversity was evaluated by principal coordinate analysis (PCoA) based on Bray–Curtis dissimilarity with the vegan package (v2.5-3). No extraction blank or PCR negative control was included in the original sequencing workflow.

Because this approach utilized 16S rRNA gene amplicon sequencing of total extracted DNA, the detected sequences may originate from both viable and non-viable bacterial cells. Consequently, the results reflect the community composition at the DNA level, rather than representing direct bacterial activity. Different bacterial samples are designated by specific letter codes. LC, HC, and NC refer to samples collected before larval inoculation in Lianyungang, Huaian, and Nanjing, respectively, whereas LT, HT, and NT represent samples collected after larval inoculation in the corresponding sites.

Potential bacterial functions were inferred from 16S rRNA gene data using the PICRUSt2 pipeline (v1.1.0). Representative OTU sequences were aligned to reference sequences, integrated into a reference phylogeny, and normalized based on predicted 16S copy numbers. Subsequently, gene family and metabolic pathway profiles were predicted following the standard PICRUSt2 workflow. It should be noted that the COG-based functional categories presented in the Results reflect predicted functional potential, rather than direct measurements of bacterial activity. Predicted gene family profiles were classified into COG functional categories for subsequent comparison among treatments. As these results were inferred from 16S rRNA amplicon data, rather than directly measured by metagenomic or metatranscriptomic sequencing, they represent only potential functional profiles. This caveat is particularly relevant for analyses of gut and leaf endophytic bacterial communities.

Since different 16S rRNA hypervariable regions were used to analyze the soil, leaf, and larval samples, direct comparisons at the OTU or ASV sequence levels were considered inappropriate. Consequently, analyses of bacterial community commonality were performed exclusively at the genus level. Genus-level data were utilized for Venn diagram analyses to assess the overlap of bacterial genera among the three study sites and across the three sample types: soil (SLT), soybean leaves (LLT), and larval midgut (ILT). The size of the core bacterial community—that is, the number of genera shared among all three sample types—is clearly indicated within the overlapping regions of each diagram.

### 2.5. Nutrition Information

#### 2.5.1. Soil Physicochemical Properties

Soil physicochemical properties were determined according to Soil Agricultural Chemical Analysis [30]. Soil pH was measured with a pH potentiometer at a soil-to-water ratio of 1: 2.5 (*w*/*v*). Total nitrogen was determined using the Kjeldahl method. Total phosphorus was analyzed by the NaOH fusion-molybdenum antimony anti-spectrophotometric method, and total potassium was measured using the NaOH fusion method. Available nitrogen was assessed using the alkaline hydrolysis diffusion method, available phosphorus by spectrophotometry, and available potassium was extracted with ammonium acetate and then quantified. Soil organic matter content was determined using the potassium dichromate volumetric method.

#### 2.5.2. Nutritional Composition of *C. bilineata tsingtauica* Larvae

All nutritional data for *C. bilineata tsingtauica* larvae were based on fresh weight. Prior to analysis, entire larvae were flash-frozen in liquid nitrogen and ground into a fine powder. Crude protein content was determined using the Kjeldahl method. Briefly, 0.2 g of each sample was placed in a digestion tube, followed by sequential addition of 0.4 g CuSO_4_, 6 g K_2_SO_4_, and 12 mL H_2_SO_4_. The mixture was digested in a digestion furnace at 420 °C until the solution became bluish-green and then clear; digestion continued for an additional hour. After cooling, 20 mL of water was added and to the mixture cooled further. Distillation was performed for 7 min using an automatic Kjeldahl nitrogen analyzer. The receiving flask contained 10 mL of boric acid solution (20 g/L) and 1–2 drops of a mixed indicator solution of methyl red and bromocresol green. Distillate was collected to a final volume of 200 mL and then titrated with 0.1 mol/L HCl to a pale grayish-red endpoint. A reagent blank was processed in parallel. Crude protein content was estimated using the Kjeldahl method, calculated as total nitrogen × 4.76 [31]. The term “crude protein” is used throughout this study because Kjeldahl nitrogen accounts for both protein-derived and non-protein nitrogenous compounds. The conversion factor of 4.76 was chosen in accordance with recommendations for edible insects to minimize overestimation compared to the conventional factor of 6.25.

For amino acid determination, 0.2 g fresh larva sample was weighted and placed into a hydrolysis tube. 10 mL of a 1:1 hydrochloric acid solution was added, thoroughly mixed, and the tube was incubated in an electric forced-air oven at 110 ± 1 °C for 22 h. After incubation, the tube was allowed to cool to room temperature. The hydrolysis tube was then opened and the hydrolysate was filtered into a 25 mL volumetric flask. The hydrolysis tube was rinsed several times with a small amount of water, and the rinses were combined with the filtrate in the flask. The mixture was diluted to volume with water and mixed well. Subsequently, 0.5 mL of the resulting solution was pipetted into a 15 mL test tube, dried under a stream of nitrogen, and reconstituted to 10 mL with 0.02 mol/L hydrochloric acid solution. After thorough mixing, the solution was filtered through a 0.22 μm microporous membrane and analyzed using the appropriate instrument. Amino acid composition was determined using an automatic amino acid analyzer (LA8080; Hitachi High-Tech Science Corporation, Tokyo, Japan) equipped with a post-column ninhydrin derivatization system and photometric detection at 570 nm and 440 nm. Seventeen hydrolyzed amino acids—aspartic acid, threonine, serine, glutamic acid, glycine, alanine, cystine, valine, methionine, isoleucine, leucine, tyrosine, phenylalanine, lysine, histidine, arginine, and proline—were quantified based on their detector responses following chromatographic separation. Tryptophan was quantified separately by high-performance liquid chromatography (HPLC) using an Agilent C18 column (4.6 mm × 150 mm, 5 μm; Santa Clara, CA, USA). The column temperature was maintained at 35 °C, with an injection volume of 10.0 μL, a flow rate of 1.2 mL/min, and UV detection at 280 nm.

During the determination of fatty acids, 0.2 g fresh larva sample was weighed into a 100 mL colorimetric tube. For hydrolysis, 2 mL of 95% ethanol and 4 mL of water were added, and the mixture was thoroughly homogenized. Subsequently, 10 mL of 8.3 mol/L hydrochloric acid solution was added, and the sample was mixed thoroughly. The tube was then placed in an 80 °C water bath for 40 min to allow hydrolysis to proceed. During hydrolysis, the tube was shaken once every 10 min to ensure that particles adhering to the wall were fully resuspended in the solution. After hydrolysis, the sample was removed from the water bath and cooled to room temperature. For fat extraction, 10 mL of 95% ethanol was added to the hydrolyzed sample and mixed thoroughly. The sample was then extracted three times with 100 mL of an ether–petroleum ether mixture. The extracts were combined in a 100 mL round-bottom flask, and the organic phase was evaporated to dryness to obtain the lipid fraction. For saponification and methyl esterification, 4 mL of 2% sodium hydroxide in methanol was added to the fat extract, and the mixture was incubated in a 45 °C water bath for 20 min. Subsequently, 4 mL of 14% boron trifluoride in methanol was added, and the mixture was further incubated in a 45 °C water bath for 20 min. After incubation, the mixture was cooled to room temperature. Then, 3 mL of n-hexane was added to a centrifuge tube, and the mixture was shaken for 2 min and allowed to stand until phase separation occurred. The upper clear phase was collected, filtered through a 0.45 μm membrane filter, and subjected to instrumental analysis. Fatty acid composition was measured using a gas chromatography-mass spectrometer (Trace1310 ISQ; Thermo, Waltham, MA, USA). Separation was performed on a TG-FAME column (50 m × 0.25 mm × 0.20 μm) with an injector temperature of 260 °C, a carrier gas flow rate of 0.63 mL/min, and a split ratio of 100: 1. MS parameters included an ion source temperature 280 °C, a transfer line temperature of 240 °C, a solvent delay of 4.00 min, electron ionization (EI), and an electron energy, of 70 eV. The temperature program was: hold at 80 °C for 1 min, increase to 160 °C at 20 °C/min and hold for 1.5 min, then increase to 230 °C at 5 °C/min and hold for 6 min. Fatty acids were identified by comparing their retention times and mass spectra with those of a chromatographically pure 35-component fatty acid methyl ester (FAME) mixture standard (Sigma-Aldrich, St. Louis, MO, USA). Quantification was carried out using the single-point external standard method. The FAME mixture standard solution was prepared at a concentration of 2 mmol/L and analyzed to determine the response factor for each target fatty acid based on its peak area. Fatty acid contents in *C. bilineata tsingtauica* larvae were subsequently calculated using the following formula:

W = C × V × N/m × k × 10^−4^ (1)

where W is content of each fatty acid in the sample (g/100 g), C is concentration of fatty acid methyl esters in the sample solution (mg/L), V is final volume of the sample solution (mL), k is conversion factor for transforming fatty acid methyl esters to fatty acids, N is dilution factor, 10^−4^ is unit conversion factor, and m is mass of the sample (g).

### 2.6. Statistical Analysis

Prior to statistical analysis, normality and homogeneity of variance were assessed using the Shapiro–Wilk test and Levene’s test, respectively. Differences among groups in soil physicochemical properties and larval nutritional variables were examined using one-way or two-way ANOVA, as appropriate. When both region and treatment were considered, they were included as fixed factors in the model. When ANOVA results indicated significant effects, group means were compared using Tukey’s honestly significant difference (HSD) test at a significance level of *p* < 0.05. For analyses involving paired samples, the paired structure was appropriately incorporated into the statistical model. Bacteriome alpha-diversity indices were compared among groups by ANOVA. Beta-diversity was assessed using principal coordinate analysis (PCoA) based on Bray–Curtis dissimilarity, and differences in community composition were tested by permutational multivariate analysis of variance (PERMANOVA) implemented in the vegan package in R. For correlation analysis, all calculations were performed using individual biological replicates (five replicates from each of the three sampling sites; *n* = 15), rather than regional mean values. To account for the compositional nature of bacterial relative abundance data, bacterial genera were centered log-ratio (CLR) transformed before analysis, whereas soil physicochemical variables and larval nutritional traits were analyzed using their original measured values. Pairwise associations among soil properties, larval nutritional components, and dominant bacterial genera were evaluated using Spearman’s rank correlation. *p* values from multiple correlation tests were adjusted using the Benjamini–Hochberg false discovery rate (FDR) method, and adjusted *p* < 0.05 was considered statistically significant. Correlation results were visualized as heatmaps.

## 3. Results

### 3.1. Differences Among the Three Study Sites in Soil Nutrient Responses

**Figure 1:**
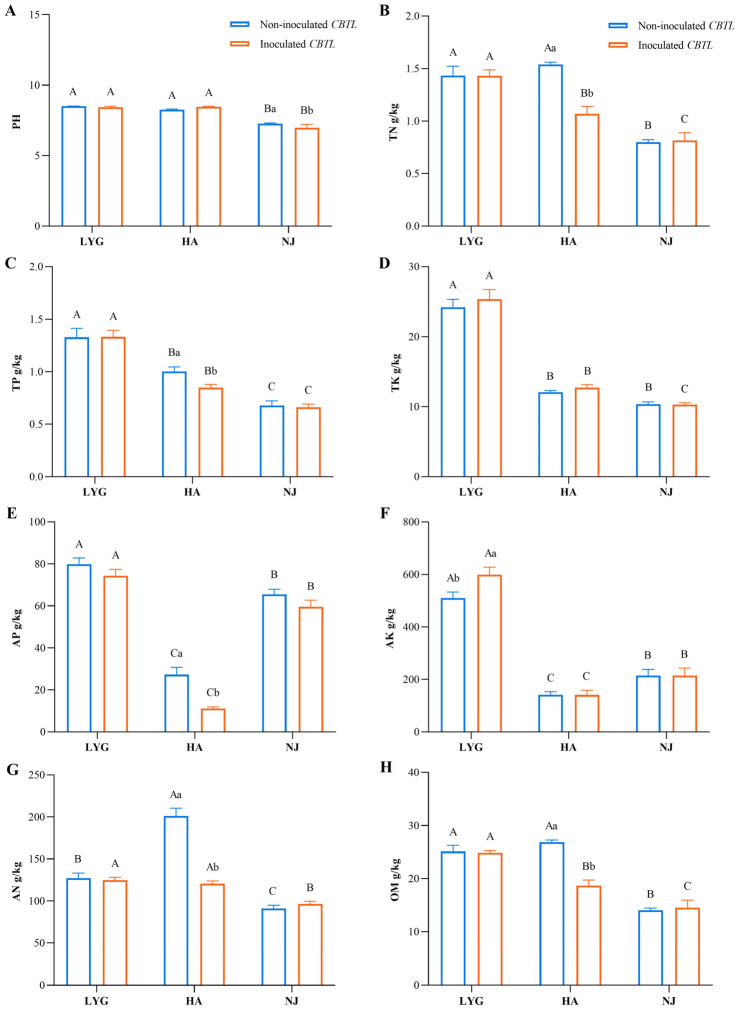
Comparison of soil nutrient contents in Lianyungang (LYG), Huaian (HA), and Nanjing (NJ). (**A**) Soil pH comparison; (**B**) Soil total nitrogen content comparison; (**C**) Soil total phosphorus content comparison; (**D**) Soil total potassium content comparison; (**E**) Soil available phosphorus content comparison; (**F**) Soil available potassium content comparison; (**G**) Soil available nitrogen content comparison; (**H**) Soil organic matter content comparison. Different letters indicate significant differences at *p* < 0.05. Uppercase letters indicate differences among regions, whereas lowercase letters indicate differences before and after larval inoculation within the same site. CBTL denotes *C. bilineata tsingtauica* larvae. The same applies to the following figures.

At the three study sites in Lianyungang (LYG), Huaian (HA), and Nanjing (NJ), we analyzed eight soil nutrient indicators for both the control group (prior to larval inoculation) and the treatment group (following larval development) (Figure 1). After inoculation with *C. bilineata tsingtauica* larvae, the soils at the three sites exhibited distinct responses: available potassium (AK) increased markedly in LYG, multiple nutrient indicators declined in HA, and NJ showed a significant reduction in soil pH. As shown in Figure 1A, soil pH in both LYG (8.5 ± 0.02 before inoculation; 8.42 ± 0.08 after inoculation) and HA (8.25 ± 0.05; 8.45 ± 0.05) was significantly higher than that in NJ (7.27 ± 0.04; 6.97 ± 0.24) both before and after inoculation. In addition, only soil pH in NJ decreased significantly after inoculation. In addition, Figure 1H indicates that, before inoculation, soil organic matter (SOM) did not differ significantly between LYG and HA, and both were significantly higher than NJ (*p* < 0.05). After inoculation, however, SOM differed significantly among the three locations, with SOM decreasing significantly in HA (*p* < 0.05). Overall, these results indicate that only NJ exhibited a significant decline in soil pH following larval inoculation. From Figure 1B,C,E,G, the contents of total nitrogen (TN), total phosphorus (TP), available nitrogen (AN), and available phosphorus (AP) decreased significantly in HA after inoculation (*p* < 0.05), suggesting an overall decline in soil nutrient status in HA after inoculation. in HA. With respect to potassium, prior to inoculation both total potassium (TK) and AK were significantly higher in LYG than in HA and NJ (*p* < 0.05) (Figure 1D,F). After inoculation, TK differed significantly among the sites (*p* < 0.05), and AK increased significantly in LYG (*p* < 0.05). These comparisons reflect changes over time within each study site, as no synchronous larva-free endpoint controls were included.

### 3.2. Differences Among the Three Study Sites in Larval Nutritional Composition

**Figure 2:**
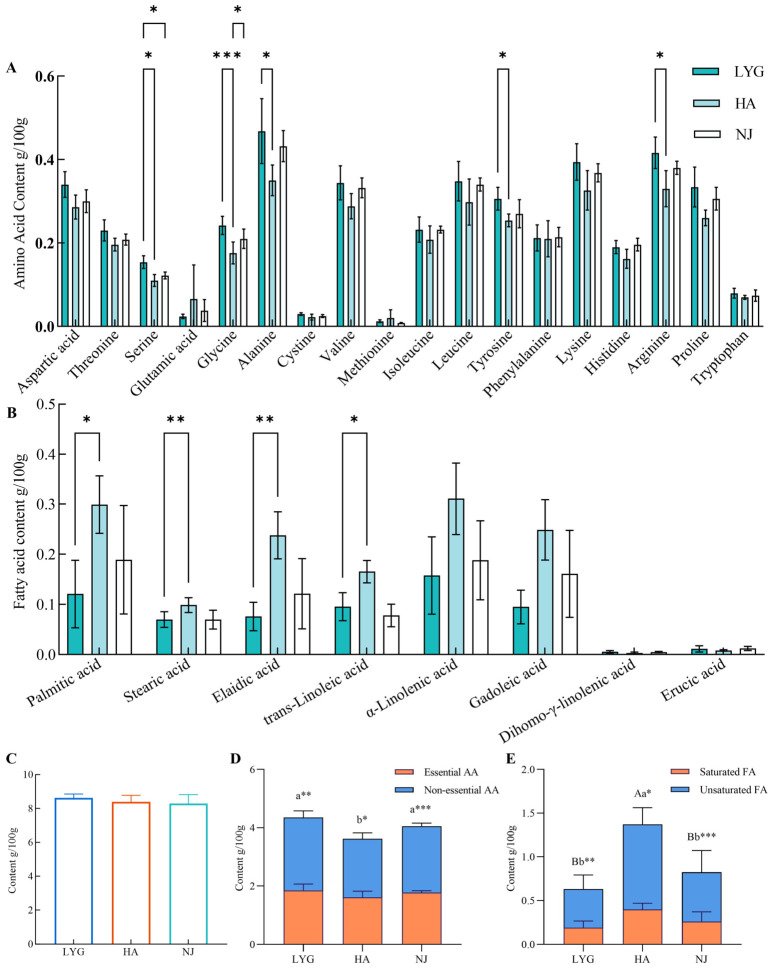
Comparison of the nutritional composition in *C. bilineata tsingtauica* larvae (CBTL). (**A**) Amino acid contents; (**B**) Fatty acid contents; (**C**) Total crude protein contents; (**D**) Essential and non-essential amino acid (AA) contents; (**E**) Saturated and unsaturated fatty acid (FA) contents. Different letters above the bars indicate significant differences among groups (one-way ANOVA followed by Tukey’s HSD test, *p* < 0.05). Uppercase letters denote essential amino acids and saturated fatty acids, whereas lowercase letters denote non-essential amino acids and unsaturated fatty acids). Asterisks indicate significant differences between categories (essential vs. non-essential amino acids; saturated vs. unsaturated fatty acids) within the same group (* *p* < 0.05; ** *p* < 0.01; *** *p* < 0.001).

The nutritional composition of *C. bilineata tsingtauica* larvae differed significantly among the three sites (Figure 2). With respect to amino acid composition (Figure 2A), among the 18 amino acids assessed, five—serine (Ser), glycine (Gly), alanine (Ala), tyrosine (Tyr), and arginine (Arg)—varied significantly (*p* < 0.05). Specifically, the concentrations of these five amino acids were higher larvae from LYG than in those from HA (*p* < 0.05). Ser content was also higher in LYG than in NJ (*p* < 0.05), whereas Gly content was higher NJ than in HA (*p* < 0.05). As shown in Figure 2D, the non-essential amino acid in HA larvae was significantly lower than that in larvae from LYG and NJ (*p* < 0.05). Although individual amino acid contents differed substantially, the total crude protein content of *C. bilineata tsingtauica* larvae did not differ significantly among the three sites (Figure 2C). Although crude protein content did not differ significantly among sites, the profiles of individual amino acids exhibited notable variation. This suggests that differences among the three study sites are more pronounced in amino acid composition than in overall crude protein levels. For fatty acids, the levels of C16: 0, C18: 0, t-C18: 1, and t,t-C18: 2 were significantly higher in HA larvae than in LYG larvae (*p* < 0.05) (Figure 2B). In addition, as shown in Figure 2E, both saturated and unsaturated fatty acid contents were significantly higher in HA larvae than in the other two sites (*p* < 0.05).

### 3.3. Bacterial Community Composition

**Figure 3:**
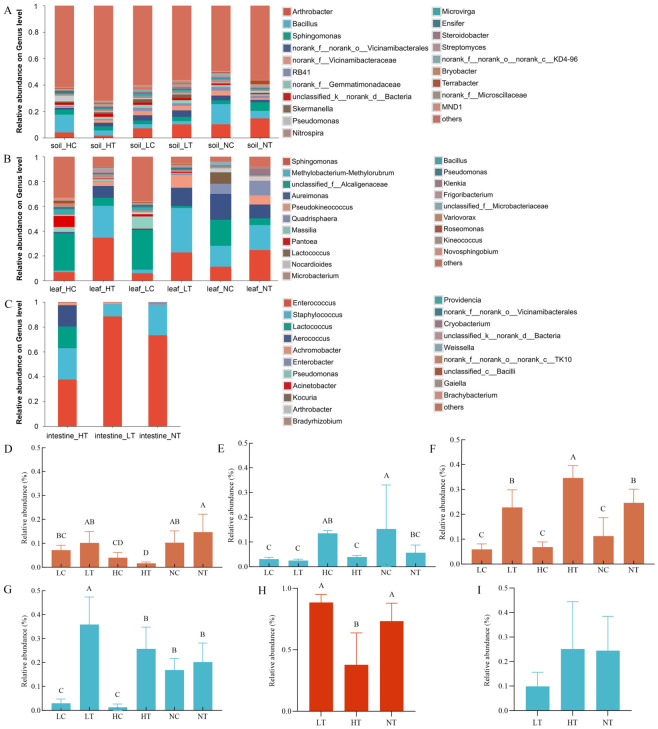
Bacterial composition in different groups of soil, leaves, and CBTL. (**A**) Relative abundance of soil bacteria at the genus level; (**B**) Relative abundance of leaf endophytic bacteriome at the genus level; (**C**) Relative abundance of CBTL bacteria at the genus level; (**D**) Proportion of *Arthrobacter* across sites; (**E**) Proportion of *Bacillus* across sites; (**F**) Proportion of *Sphingomonas* across sites; (**G**) Proportion of *Methylobacterium-Methylorubrum* across sites; (**H**) Proportion of *Enterococcus* across sites; (**I**) Proportion of *Staphylococcus* across sites. Data in panels (**D**–**I**) are presented as mean ± SD (*n* = 6 biological replicates). Different uppercase letters above the bars indicate significant differences among groups at *p* < 0.05. In the x-axis labels, L, H, and N represent Lianyungang, Huaian, and Nanjing, respectively; whereas C and T indicate before and after larval inoculation, respectively. Midgut samples were collected exclusively after larval inoculation; therefore, only group T is shown for the CBTL midgut samples.

Genus-level analysis of the bacterial communities in soil, soybean leaves, and larval midgut samples from Lianyungang (LYG), Huaian (HA), and Nanjing (NJ) showed distinct regional patterns. Figure 3A–C show the genus-level patterns of bacterial composition in soil, leaves, and CBTL; data at the replicate level are included in Supplementary Figure S2. As shown in Figure 3A,D,E, *Arthrobacter* and *Bacillus* were the dominant bacterial genera in the soil across all three sites. *Arthrobacter* abundance was highest in NT soil and lowest in HT soil, whereas *Bacillus* abundance was highest in NC soil and lowest in LC, LT, and HT soils. *Sphingomonas* and *Methylobacterium-Methylorubrum* were the dominant genera in the leaves across the three sites. As shown in Figure 3B,F,G, *Sphingomonas* abundance in leaves before inoculation was significantly lower than that after inoculation in all three sites (*p* < 0.05). Figure 3C,H,I indicate that *Enterococcus* and *Staphylococcus* were the dominant bacterial genera in the larval gut across the three sites. The relative abundance of *Enterococcus* was higher in LT and NT than in HT (*p* < 0.05), whereas *Staphylococcus* did not differ significantly among sites (*p* > 0.05).

### 3.4. Genus-Level Taxonomic Overlap Among Soil, Leaves, and Larvae

**Figure 4:**
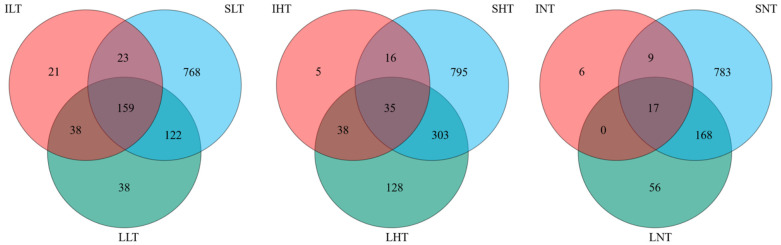
Venn diagram showing genus-level overlap of bacterial communities among soil, soybean leaf, and CBTL gut samples across the three study sites. Red, blue, and green circles represent CBTL gut, soil, and soybean leaf samples, respectively. The numbers in the non-overlapping and overlapping regions indicate the numbers of unique and shared bacterial genera, respectively. The three panels correspond to the Lianyungang, Huaian, and Nanjing sites, respectively. In the sample abbreviations, I, S, and L denote insect gut, soil, and leaf samples, respectively, and LT, HT, and NT indicate Lianyungang, Huaian, and Nanjing, respectively.

To avoid inappropriate direct comparisons of amplicon-defined OTUs generated from different 16S rRNA regions, cross-compartment overlap was assessed at the genus level following taxonomic annotation. The analysis indicated partial genus-level overlap among bacterial communities in soil, soybean leaves, and larval guts, but did not demonstrate direct transmission or source attribution. Within each study site, the number of shared bacterial genera generally decreased from soil to leaves to larvae (Figure 4). Among the genera present in all three compartments (soil, leaves, and larvae), LYG exhibited the highest number (159 genera), while NJ had the lowest (17 genera). HA showed the highest bacterial richness in soybean leaves (504 genera). However, the number of genera shared between leaves and larvae was lower in HA than in LYG, and lowest in NJ (17 genera). Additionally, several bacterial genera were detected exclusively in soil and larval gut samples but not in soybean leaves across all three sites. These results indicate a conservative genus-level overlap among compartments and sites, but should not be regarded as definitive evidence of bacterial transmission pathways.

### 3.5. Bacterial Diversity and Community Structure

**Figure 5:**
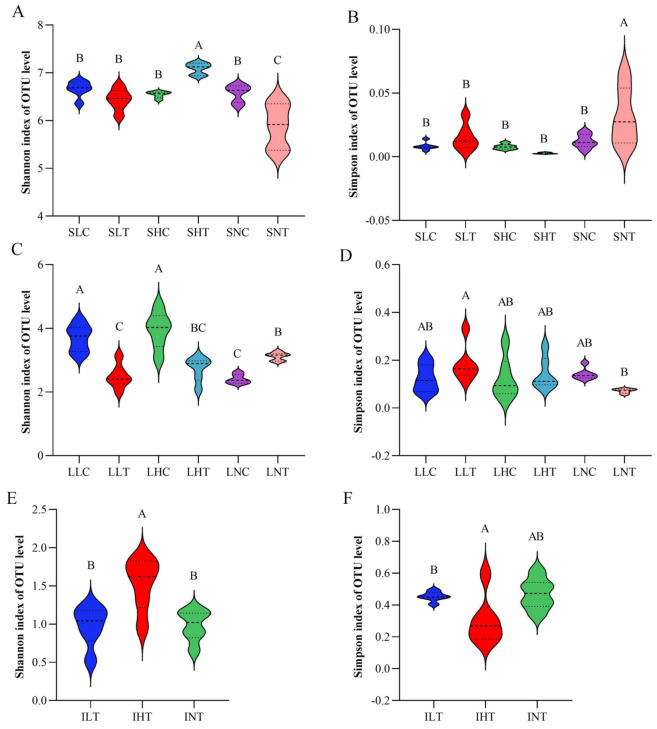
Alpha-diversity analysis of bacterial communities at the OTU level in soil, leaves, and *C. bilineata tsingtauica* larval gut. (**A**) Shannon diversity index at the soil OTU level; (**B**) Simpson diversity index at the soil OTU level; (**C**) Shannon diversity index at the leaf OTU level; (**D**) Simpson diversity index at the leaf OTU level; (**E**) Shannon diversity index at the *C. bilineata tsingtauica* larval OTU level; (**F**) Simpson diversity index at the *C. bilineata tsingtauica* larval OTU level. In site abbreviations, L, H, and N indicate Lianyungang, Huaian, and Nanjing, respectively. C and T indicate samples collected before and after larval inoculation, respectively. Thus, LC, HC, and NC refer to pre-inoculation samples from the three sites, whereas LT, HT, and NT refer to post-inoculation samples. For larval gut samples, ILT, IHT, and INT indicate larval guts collected from Lianyungang, Huaian, and Nanjing, respectively. Different uppercase letters above the violins indicate significant differences among groups at *p* < 0.05. The dashed lines within each violin indicate the median and quartiles.

**Figure 6:**
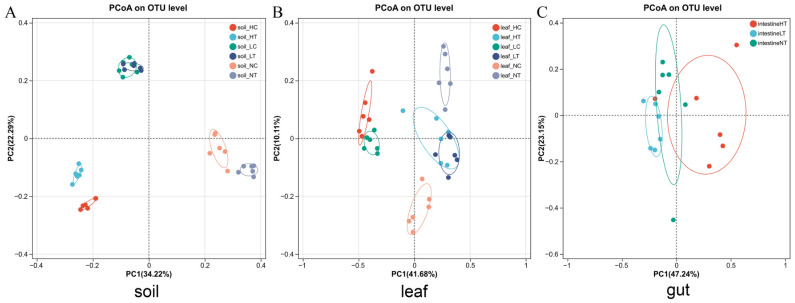
Beta-diversity analysis of bacterial communities at the OTU level in soil, leaves, and *C. bilineata tsingtauica* larval gut. (**A**) Principal coordinate analysis (PCoA) at the soil OTU level; (**B**) PCoA at the leaf OTU level; (**C**) PCoA at the *C. bilineata tsingtauica* larval gut OTU level. In site abbreviations, L, H, and N indicate Lianyungang, Huaian, and Nanjing, respectively. C and T indicate samples collected before and after larval inoculation, respectively. Thus, LC, HC, and NC refer to pre-inoculation samples from the three sites, whereas LT, HT, and NT refer to post-inoculation samples.

Based on genus-level analysis, *α*-diversity and *β*-diversity of the bacterial communities in soil, leaves, and the midgut of *C. bilineata tsingtauica* larvae from the three sites were assessed (Figure 5 and Figure 6). As shown in Figure 5A, the Shannon index in the SHT group was significantly higher than those in SLC, SLT, SHC, SNC, and SNT (*p* < 0.05), whereas the Shannon index of SNT was significantly lower than those of the five groups mentioned above (*p* < 0.05). The Simpson index of SNT was significantly higher than those of SLC, SLT, SHC, SHT, and SNC (*p* < 0.05) (Figure 5B). Principal coordinate analysis (PCoA) based on the relative abundance of soil bacterial communities (Figure 6A) showed that PC1 and PC2 accounted for 34.22% and 22.29% of the variation, respectively, with a cumulative contribution of 56.51%. Except for SLC and SLT, the samples of SHC, SHT, SNC, and SNT were broadly dispersed in the two-dimensional space, indicating pronounced differences in soil bacterial community structure among these treatments.

For the leaf groups, Figure 5C indicates that the diversity of leaf endophytic bacteriome generally decreased after larval inoculation in LYG and HA. The Shannon indices of LLC and LHC were significantly higher than those of other treatment groups (*p* < 0.05). In addition, the Simpson index of LLT was significantly higher than that of LNT (*p* < 0.05) (Figure 5D). Meanwhile, PCoA based on relative abundance (Figure 6B) placed LLC and LHC in close proximity but without overlap, suggesting broadly similar community structures with considerable differences.

**Figure 7:**
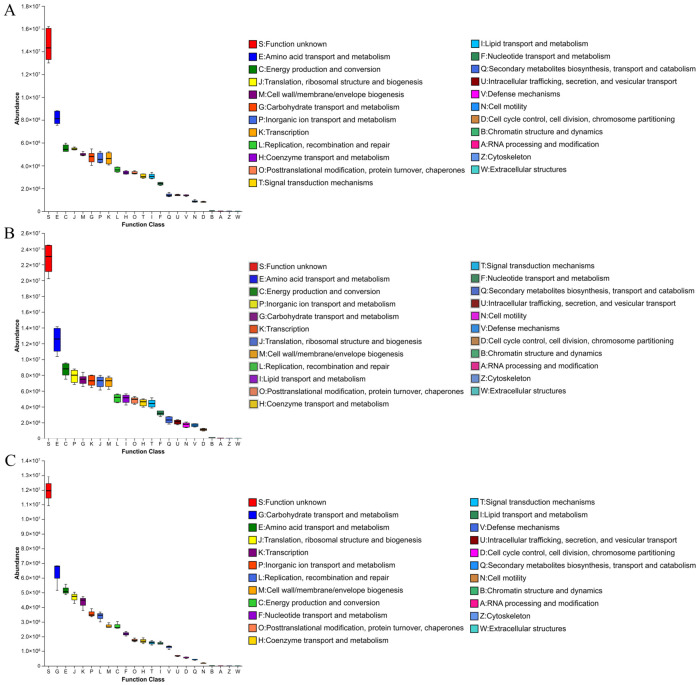
Predicted COG functional profiles of bacterial communities inferred by PICRUSt2. (**A**) Soil bacterial community; (**B**) soybean leaf endophytic bacterial community; (**C**) larval gut bacterial community.

For the larval groups, Figure 5E shows that the Shannon index of IHT was significantly higher than those of ILT and INT (*p* < 0.05). In Figure 5F, the Simpson index of IHT was significantly higher than that of ILT (*p* < 0.05). PCoA based on relative abundance (Figure 6C) demonstrated that both ILT and IHT partially overlapped with INT, whereas was observed between ILT and IHT, indicating distinctiveness between these two treatment groups. To further compare the predicted functional potential of bacterial communities across different compartments and sites, PICRUSt2-based inference was conducted and summarized according to Clusters of Orthologous Groups (COG) functional categories (Figure 7). As shown in Figure 7A–C, the rhizosphere soil bacteria, leaf endophytic bacteria, and larval gut bacteria exhibited distinct predicted functional profiles. Since these results are based on 16S rRNA data rather than direct measurements of bacterial activity, they represent inferred functional potential and should be interpreted with caution.

### 3.6. Correlations Among Soil Nutrients, Bacteria, and Larval Nutrition

**Figure 8:**
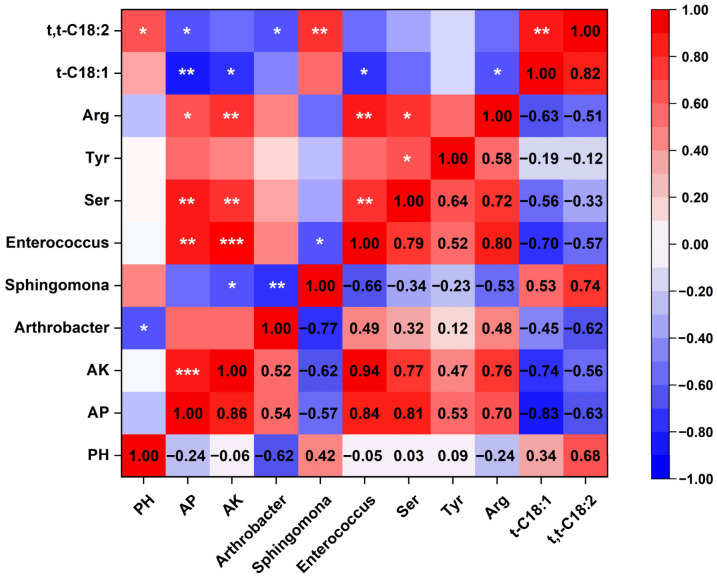
Correlation heatmap of soil physicochemical properties, larval nutritional components, and dominant bacterial genera. Correlation analyses were performed using individual biological replicates (*n* = 15) and assessed with Spearman’s rank correlation. Bacterial relative abundance data were centered log-ratio (CLR) transformed prior to analysis. Asterisks denote statistically significant Spearman correlations after Benjamini–Hochberg (BH) false discovery rate (FDR) correction (* adjusted *p* < 0.05, ** adjusted *p* < 0.01, *** adjusted *p* < 0.001).

Correlation analysis was performed to examine associations among soil physicochemical properties, the nutritional components of *C. bilineata tsingtauica* larvae, and dominant bacterial genera. Correlation analysis identified several significant associations among soil properties, larval nutritional components, and dominant bacterial genera across the various niches. All analyses were conducted using individual biological replicates (*n* = 15). To minimize compositional bias, bacterial relative abundance data were transformed using the centered log-ratio (CLR) method prior to analysis. Pairwise associations were then evaluated with Spearman’s rank correlation, and statistical significance was adjusted using the Benjamini–Hochberg false discovery rate (FDR) correction. As shown in Figure 8 several significant associations were detected among soil variables, larval amino acids and fatty acids, and dominant bacteria. In general, Soil pH was significantly positively correlated only with t,t-C18:2 (ρ = 0.68 *), and significantly negatively correlated with *Arthrobacter* (ρ = −0.62 *). AP, AK, and *Enterococcus* all showed highly significant positive correlations (AP vs. *Enterococcus*, ρ = 0.84 **; AK vs. *Enterococcus*, ρ = 0.94 ***). AP, AK, and Ser showed significant positive correlations (AP-Ser ρ = 0.81 **; AK-Ser ρ = 0.77 **). AK showed a significant negative correlation with *Sphingomonas* (ρ = −0.62 **). AP was significantly negatively correlated with both fatty acids: AP and t-C18:1 had a ρ value of −0.83 **, AP and t,t-C18:2 had a ρ value of −0.63 *. AK and t-C18:1 had a ρ value of −0.74 *. AP and AK showed a significant positive correlation with Arg (AP-Arg ρ = 0.70 *; AK-Arg ρ = 0.76 **). *Enterococcus* was significantly negatively correlated with *Sphingomonas* (ρ = −0.66 **), while *Arthrobacter* was significantly negatively correlated with *Sphingomonas* and t,t-C18:2 (−0.77 ** and −0.62 *). *Sphingomonas* showed a significant positive correlation with t,t-C18:2 (ρ = 0.74 **). *Enterococcus* was significantly positively correlated with Ser and Arg (0.79 ** and 0.80 **). All three amino acids showed significant positive correlations with one another: Ser-Tyr ρ = 0.64 **, Ser-Arg ρ = 0.72 **, Tyr-Arg ρ = 0.58 *. Arg was significantly negatively correlated with t-C18:1 (ρ = −0.63 *). t-C18:1 showed a significant positive correlation with t,t-C18:2 (ρ = 0.82, **). Overall, these results indicate that soil properties, larval nutritional traits, and bacterial genera are interconnected through significant associations. However, these correlations do not imply direct causal relationships, and the underlying mechanisms require further experimental validation.

## 4. Discussion

This study investigated soil physicochemical properties, the nutritional composition of *C. bilineata tsingtauica* larvae and 16S-based bacteriome profiles in soil, leaves, and larvae across different sites, and further examined the associations of soil properties and bacterial compositions with larval nutritional traits. The results showed that larval inoculation was accompanied by significant changes in several soil nutrients across the three sites. In parallel, the contents of five amino acids (Ser, Gly, Ala, Tyr, Arg) and four fatty acids (C16: 0, C18: 0, t-C18: 1, t,t-C18: 2) in larvae were correlated to varying degrees, with soil nutrients and bacterial genera. Collectively, these findings suggest that regional soil background conditions are connected with the soil–soybean–larva system and may therefore influence bacterial assembly and larval nutrient metabolism.

At the soil level, the presence of *C. bilineata tsingtauica* larvae was related to complex and site-specific responses in the soil–plant system. These differences may reflect the combined influence of initial soil conditions, larval metabolic activity, and plant nutrient uptake. When changes in soil pH and SOM were considered together, only NJ soil showed a significant decrease in pH after larval inoculation. This pattern may be related to the lower SOM content in NJ, because soil organic matter is an important buffering component that contributes to pH stability [32]. The lower SOM content in NJ may therefore have reduced buffering capacity and cause a significant decrease in soil pH associated with larval activity [33]. In HA, total nitrogen (TN), total phosphorus (TP), available nitrogen (AN), and available phosphorus (AP) all decreased significantly after larval inoculation (*p* < 0.05), suggesting an overall decline in soil nutrient status. One possible explanation is that vigorous plant growth accelerated the consumption of available soil nutrients [34]. Meanwhile, the observed declines in certain soil nutrient pools may result from a combination of plant uptake, larval feeding, and nutrient assimilation [35]. However, as this study did not include a soybean-only control group without larvae inoculation, the relative contribution of larval activity cannot be separated directly from plant-driven nutrient depletion. Consequently, this interpretation should be considered hypothetical rather than a confirmed mechanism. In contrast to the depletion pattern in HA, available potassium (AK) increased significantly in LYG soil after larval inoculation. One possible explanation is the input of frass, as insect excreta can be enriched in mineral elements such as potassium [36]. However, plant age, seasonal changes, and natural rhizosphere development may also have contributed to the observed variation in soil nutrient levels and bacteria composition. Due to the absence of a soybean-only control group, the potential influence of region-specific plant uptake dynamics or other soil processes cannot be excluded. As this study was conducted under field conditions across different geographic locations, some degree of environmental heterogeneity is unavoidable. Consequently, the observed variations should be interpreted as site-specific soil responses to larval inoculation, rather than as definitive evidence of a direct causal relationship.

These differences among the three study sites in soil nutrient dynamics may, in turn, help explain the nutritional divergence observed in larvae. Because larvae were collected from a shared inoculated plot and could move among plants, replicate independence at the single-plant level cannot be assumed. Overall, larvae from LYG showed the highest amino acid levels, whereas those from HA had relatively lower levels. When the physicochemical characteristics of the two sites were compared, TP, TK, AP, and AK were all significantly lower in HA soil than in LYG soil. Since soybean leaves serve as a direct food source for the larvae of *C. bilineata tsingtauica*, a plant-mediated pathway is biologically plausible. However, this study did not directly assess the nutritional composition of the leaves. As a result, it is not possible to determine whether differences among the sites in the larvae’s amino acid profiles are mediated by variations in soybean protein, free amino acids, or other nutritional components. Therefore, our interpretation is limited to the possibility that regional soil nutrient conditions may have indirectly influenced the larvae’s nutritional status by affecting the host plant [37]. Previous studies have demonstrated that soil nutrient availability influences the nutritional quality of plants [38,39]; however, this mechanism has not yet been directly verified in our research system. Notably, although individual amino acid contents varied substantially among sites, total crude protein content did not differ significantly, highlighting the metabolic homeostasis of insects under heterogeneous environmental conditions [40]. It should be noted that the Kjeldahl method estimates crude protein based on total nitrogen content and may overestimate true protein levels, as insect tissues contain non-protein nitrogen sources, such as chitin-associated nitrogen. Although the insect-specific conversion factor of 4.76 was employed to mitigate this bias, the reported values should be interpreted as estimates of crude protein rather than absolute true protein content. The use of alternative methods, such as spectroscopic techniques, may provide more accurate protein quantification. Nevertheless, because the same protocol was consistently applied to all regional samples, this approach remains suitable for relative comparisons among treatment groups. In addition, crude protein content and the sum of individual amino acids were determined using different analytical methods and are therefore not directly comparable. As previously noted with respect to protein determination methods, the actual amount of amino acid residues derived from protein may be overestimated. In contrast, amino acid content was measured by quantifying individual amino acids after hydrolysis; this sum may be lower because some amino acids can be partially degraded during hydrolysis or may be recovered with lower efficiency. Accordingly, the relatively consistent crude protein content observed across the sites is not inconsistent with differences among the sites in amino acid composition or with lower total amino acid yields. More broadly, the larval nutritional profiles revealed clear regional differentiation: LYG larvae had the highest levels of several amino acids (Ser, Ala, Gly, Arg, and Tyr), whereas HA larvae showed a distinct “high-fatty-acid, low-amino-acid” phenotype (C16: 0, C18: 0, t-C18: 1, t,t-C18: 2). Together with the differences among the three study sites in soil nutrients and the apparent trade-off between amino acids and fatty acids, these results suggest that *C. bilineata tsingtauica* larvae may adopt different resource allocation strategies under different soil environments [41]. Such allocation patterns may be mediated, at least in part, by host soybean plants. Accordingly, we hypothesize that variation in specific chemical constituents of soybean plants grown under different soil conditions may contributes to the observed differences in larval nutritional composition [27].

In addition to the direct effects of soil nutrients, the bacterial response to larval inoculation may represent another important pathway linking soil environment and larval metabolism. When interpreted together with soil pH and SOM, the bacterial data suggest that shifts in edaphic conditions were accompanied by shifts in key bacterial genera. Because the primer sets and target regions used for leaf samples differed from those used for soil and gut samples, bacterial community comparisons were performed within each sample type, and direct taxonomic comparisons across sample types should be interpret-ed with caution. In HA, SOM decreased significantly after larval inoculation, whereas NJ showed significant decrease in soil pH. Under these conditions, *Arthrobacter* showed a decreasing trend in HA but an increasing trend in NJ, while *Bacillus* decreased significantly in both HA and NJ after inoculation. These patterns suggests that changes in soil organic matter and pH may have contributed to the responses of these bacterial genera [42], although other unmeasured soil properties could also have been involved. The decline of both *Arthrobacter* and *Bacillus* under reduced SOM is consistent with the idea that organic matter is an important energy source for bacterial growth and reproduction [43]. By contrast, the differing responses of *Arthrobacter* and *Bacillus* to pH changes imply taxon-specific sensitivities, and may indicate that the Bacillus populations present in NJ were particularly responsive to a decrease in soil pH [44]. Following larval inoculation, the levels of *Sphingomonas* and *Methylobacterium-Methylorubrum* increased significantly. These two genera are involved in plant-microbe interactions in other plant systems [45], but their functions were not directly tested in this study. However, because plant physiological status and defense metabolites were not measured, this interpretation remains speculative. In larvae, *Enterococcus* was identified as the dominant gut associated bacterium. Previous studies have indicated that *E. casseliflavus* can be involved in larval amino acid metabolism, including pathways related to alanine in other experimental systems [28]. This is generally consistent with the lower-amino-acid concentrations observed in HA larvae. Notably, we found that higher fatty acid levels co-occurred with lower *Enterococcus* abundance in HT larvae, whereas higher amino acid levels coincided with higher *Enterococcus* abundance in LT and NT larvae. These patterns suggest a potential association between *Enterococcus* abundance and variation in larval amino acid and fatty acid profiles [46]. However, this interpretation remains hypothetical and is not indicative of a causal relationship. As our findings are based on correlations between relative bacterial abundance and nutrient levels, it remains unclear whether *Enterococcus* directly influences host metabolism, responds to host nutritional status, or simply co-varies with other environmental and microbial factors. Further investigation, including targeted functional assays, bacterial isolation, and controlled feeding or inoculation experiments, will be necessary to test these hypotheses and clarify the role of *Enterococcus* in larval metabolic processes.

To better understand how these bacterial differences arise, it is useful to consider possible routes of bacterial acquisition and compartmental overlap across the soil–leaf–larva system. The overlap analysis showed that the number of shared bacterial genera generally decreased from soil to leaf to larva, and that regional variation was present throughout this system. This pattern is consistent with compartment-specific filtering, but does not by itself demonstrate directional transmission. Some bacterial genera may be connected with the host plant, whereas others may reach larvae through alternative routes, including leaf-surface contamination, ingestion of soil- or dust-associated particles, frass contamination, air exposure, contact with substrate, or interactions among larvae [47]. Such filtering may occur because bacteria colonize different plant compartments during transmission, with some remaining in roots or stems and failing to migrate upward [48]. After entering the larval body, some bacteria may be unable to adapt to the gut environment and die, whereas others may colonize only transiently and then be excreted, resulting in lower bacterial richness in larvae than in leaves [49]. The lower bacterial richness observed in larvae compared to leaves may therefore result from both ecological filtering and the transient passage of environmentally acquired bacteria. Similarly, the presence of bacterial genera detected only in soil and larval gut samples may indicate unsampled plant compartments, external contamination routes, or direct environmental acquisition, rather than a straightforward soil–leaf–larva transmission pathway [50]. Given that this study relied on field comparisons and DNA-based community profiling without the use of source-tracking models, strain-level resolution, or controlled transmission assays, these findings should be interpreted as evidence of taxonomic overlap and potential acquisition routes, rather than as definitive proof of bacterial transmission.

It is particularly noteworthy that, across all three sites, several bacterial genera were consistently detected in both soil and larval gut samples but were absent from soybean leaves. This pattern suggests that larval bacterial acquisition cannot be fully explained by the leaf-associated bacterial data obtained in this study. Potential explanations include direct environmental acquisition, leaf-surface contamination, unsampled plant compartments, or transient passage through the gut. These findings therefore indicate a compartmentalized and region-specific system of bacterial overlap, rather than a strictly linear transmission pathway from soil to leaves to larvae. Future research incorporating source-tracking methodologies, strain-level identification, and controlled exposure experiments will be necessary to elucidate the true origins and transmission routes of these bacteria. Another important factor to consider is the physiological stress imposed on plants by larval feeding. Herbivory can alter host plant growth, tissue quality, and the activation of defensive signaling pathways, including the induction of secondary metabolites such as jasmonic acid, phenolic compounds, and other defense-related chemicals [51]. These plant responses may, in turn, influence leaf-associated bacterial communities as well as the larval feeding environment. However, since we did not directly assess the health, growth characteristics, or defense-related metabolites of the inoculated soybeans, we cannot determine whether the observed differences in leaf bacterial communities and larval nutritional composition were primarily driven by soil background, plant physiological responses to herbivory, or interactions among these factors. Future studies should directly quantify soybean secondary metabolites and antinutritional factors, in order to test the proposed soil–soybean–larva linkage more rigorously.

The diversity analyses further support the view that regional edaphic context and larval activity jointly influence bacterial community assembly. Combined with soil nutrient patterns, the higher Shannon index in SHT may be linked to the relatively higher soil fertility in HA, although additional unmeasured soil properties may also have contributed [52]. By contrast, the gradual decrease in soil pH in NJ soil after larval inoculation may have restricted some bacterial groups, favoring dominance by fewer genera and thereby increasing the Simpson index [53]. Conversely, reduced bacterial diversity may also have weakened soil buffering capacity and may contributed to a decrease in soil pH in NJ [54], suggesting that the relationship between pH and diversity may be bidirectional. Principal component analysis showed partial overlap between SLC and SLT, indicating that the soil bacterial community in LYG may have greater resistance or resilience to larval-associated disturbance, as larval activity did not markedly alter the local soil bacterial composition. The diversity changes observed in HA and NJ further support the existence of region-specific bacterial responses. Larval feeding and excretion may affect soil bacteria through changes in root exudation patterns and soil nutrient dynamics [55]. In leaves, endophytic bacterial diversity generally decreased after larval inoculation in LYG and HA, suggesting that herbivory may reduce the richness of leaf associated bacterial communities. This is consistent with the conclusion of Humphrey (2014) that herbivorous insect feeding can alter plant bacterial community composition [56]. The significant differences among LLC, LHC, and LNC further indicate strong regional effects on leaf bacterial communities. Although LLC and LHC were closely positioned in ordination space, they did not overlap, indicating broadly similar but still distinguishable community structures. This suggests that larval introduction did not cause uniform shifts in leaf endophytic bacteriome, but rather than regional background conditions constrained the direction and magnitude of change. Although regional factors influenced endophytic bacteria population structure in LYG and HA, soybean plants in both sites may still have selected bacterial genera with similar functions or resistance traits in response to larval infestation as a biotic stress [57]. In NJ the lack of overlap between LNC and LNT, together with the significant change in Shannon index, suggests a stronger shift in the leaf endophytic bacteriome following larval inoculation. This shift may reflect feeding damage, altered plant physiology, or herbivory-induced changes in leaf chemistry [58], but these mechanisms were not directly tested here. In the larval gut, ILT and IHT partially overlapped with INT, whereas ILT and IHT did not overlap with each other, indicating both shared and region-specific bacterial components. This suggests that, despite geographical distance, NJ larvae shared some genera with larvae from LYG and HA. Given that LYG is the origin of *C. bilineata tsingtauica* farming and a major trading center in China, it is possible that larval stock movement influenced the bacteria of NJ populations. In addition, the geographic proximity between HA and NJ may have contributed to similarities in soil and plant-associated bacterial pools that were subsequently transmitted through the soil–soybean–larva system. It should be noted that the COG profiles reported here were inferred from 16S rRNA amplicon data using PICRUSt2 and therefore represent predicted functional potential rather than direct measurements of community function. Such inference is especially limited for gut and endophytic bacterial communities and should be interpreted with caution.

Taken together, the correlation analysis revealed a structured pattern of associations among soil properties, dominant bacterial genera, and larval nutritional traits. For instance, available phosphorus (AP) and available potassium (AK) were positively correlated with the abundance of *Enterococcus* and larval serine (Ser) and Arginine (Arg), while both AP and AK showed negative associations with t-C18:1. *Enterococcus* abundance was positively correlated with Ser and Arg, but negatively associated with t-C18:1. Larval Ser showed a significant positive correlation with AP, AK, and *Enterococcus*, while larval t-C18:2 showed significant positive correlations with pH and *Sphingomonas*. These patterns suggest that soil nutrient status, bacterial community structure, and larval nutrient composition may covary within the studied system. Several non-exclusive mechanisms may underlie these associations. One possibility is that variations in soil AP and AK are linked to changes in soybean nutritional status, which, in turn, may affect the amino acid and fatty acid composition in larvae. Alternatively, soil nutrients and bacterial genera may covary environmentally and be simultaneously encountered by larvae during feeding and movement, thereby influencing gut-associated bacterial composition and host nutritional traits. The negative associations between the two fatty acids and AP and *Enterococcus* may also reflect differences in larval metabolic allocation under varying environmental conditions. Nonetheless, as this analysis is correlation-based, these interpretations remain speculative. Direct evidence linking soil properties, soybean chemistry, bacterial colonization, and larval metabolism will require targeted experimental validation. Under such conditions, AP and AK may be involved in bacterial growth and bacterial protein or amino acid synthesis, which could then be digested and assimilated by the larvae [59]. The negative relationships between fatty acids and both nutrients and bacteria may reflect host physiological regulation. Ingestion of soil particles and bacteria could alter gut bacterial structure and abundance, potentially eliciting host immune or metabolic responses that affect lipid metabolism. In this context, Liu Jiaming et al. (2026) reported that butyrate produced by gut commensals can act on the fat body to activate glycerolipid and arachidonic acid metabolism through GPR41 receptor activation and histone deacetylase inhibition [46]. Thus, the differences among the three study sites in larval fatty acids observed here may be linked to variation in gut bacterial abundance and associated host responses. At the same time, plant secondary metabolites may also influence endosymbiotic bacteria during larval feeding [51,60]. Further analysis of specific genera supports the possibility of taxon-dependent effects. *Arthrobacter*, the dominant soil bacterium, was significant negative correlated with *Sphingomonas* and t,t-C18: 2. *Sphingomonas*, the dominant leaf bacterium, was significantly positively correlated with t,t-C18: 2. These patterns suggest that soil AP may simultaneously influence soybean growth and specific soil bacteria, while changes in plant internal conditions may affect the suitability of leaf tissues for *Sphingomonas*. Once such bacteria enter the larval gut, they may influence host metabolic processes either directly or indirectly through immune or bacterial interactions. Additionally, *Enterococcus*—the predominant bacterium in the larvae’s gut—exhibited significant positive correlations (*p* < 0.01) with soil available phosphorus (AP) and available potassium (AK), as well as with serine (Ser) and arginine (Arg) in the larvae. Conversely, *Enterococcus* showed significant negative correlation (*p* < 0.05) with *Sphingomonas*, the major leaf-associated bacterium, and with trans-octadecenoic acid (t-C18:1) in larvae. These relationships do not demonstrate direct substrate use or active metabolic regulation. More conservatively, they indicate that *Enterococcus* abundance co-varied with larval nutrient traits and environmental variables under field conditions. Whether *Enterococcus* contributes functionally to these patterns remains to be tested experimentally.

This study has several limitations. First, only a single site was sampled within each site. Consequently, geographic location is confounded with site-specific background factors, such as soil history, microclimate, and local management practices. Therefore, observed differences should be interpreted as site-associated patterns across three representative locations, rather than as strictly replicated regional effects. Furthermore, this study did not assess several potentially important soil parameters, including soil classification, electrical conductivity, exchangeable sodium, texture, and bulk density. Because these variables influence soil water-holding capacity, aeration, salinity status, and the structure of bacterial habitats, their omission limits the comprehensiveness of our interpretation of cross-regional soil-bacteriome interactions. In addition, this study lacked a soybean-only control treatment without larval inoculation. Consequently, changes observed in soil nutrient levels following inoculation cannot be attributed solely to larval activity, as plant uptake and background soil processes may also have contributed. Future experiments incorporating matched controls without larvae will be necessary to distinguish the individual impacts of soybean growth, larval feeding, and frass deposition on soil nutrient dynamics. In addition, we did not directly quantify soybean leaf nutritional components (such as proteins, amino acids, and fatty acids), plant growth status, or defense-related metabolites after larval inoculation. Because the host plant is the biological intermediary between soil conditions and larval feeding, the absence of these measurements limits our ability to determine whether the observed differences among the three study sites in larval nutritional traits and leaf bacteriome composition were mediated by plant nutritional quality, herbivory-induced stress responses, secondary metabolites, or their interactions. An additional limitation is that larval survival to the fifth instar was not quantified on a per-plant basis, as larvae were able to move among neighboring soybean plants after hatching. Consequently, larval samples could not be definitively linked to individual source plants, and replicate independence should therefore be interpreted at the level of the inoculated plot rather than the individual plant. Furthermore, although the inoculation density of 50 eggs per plant is consistent with current field-rearing practices, it may have influenced soybean physiology as well as associated bacterial communities. Furthermore, this study focused solely on larval nutritional composition and gut bacteria, without quantitatively assessing larval survival rates or growth performance. This limitation restricts our ability to fully elucidate the relationship between bacterial community variation and larval rearing outcomes. Furthermore, bacterial community analysis in this study was conducted using DNA-based 16S rRNA gene amplicon sequencing, which cannot distinguish between viable and non-viable bacteria or directly measure bacterial metabolic activity. As a result, the relationships identified should be interpreted as compositional associations rather than direct evidence of active bacterial function. Future work should integrate controlled feeding experiments, soil parameters, soybeans (control without larvae, leaf nutritional content), bacterial manipulation (such as cultivation and isolation, strain-level validation, transcriptomics, etc.), metabolomics, and functional assays, while incorporating phenotypic indicators such as larval survival rates and growth performance, to more directly and comprehensively test the proposed soil–soybean–larva system mechanism.

## 5. Conclusions

This study showed that regional soil background was correlated with differences in soil nutrient responses, soybean-associated bacterial communities, larval gut bacteria, and larval nutritional composition in *C. bilineata tsingtauica*. Site-specific patterns were evident in both soil properties and larval nutrient profiles, with amino acid enrichment in Lianyungang larvae and higher fatty acid accumulation in Huaian larvae. Bacterial communities differed across soil, leaves, and larvae, and genus-level overlap suggested compartmental filtering rather than a simple linear transmission pathway. Correlation analysis further indicated that there were significant associations between available phosphorus (AP), available potassium (AK), dominant bacterial genera, and the nutritional traits of the larvae. Overall, the findings support a region-dependent linkage across the soil–soybean–larva system, but these relationships should be interpreted as correlations rather than demonstrated mechanisms. Experimental validation will be required to test causality and microbial function.

## Data Availability

The OTU/ASV abundance tables, taxonomy annotations, sample metadata, raw soil property data, larval nutrient composition data, and analysis scripts/workflow are available in the Supplementary Materials and have been deposited in the Zenodo repository (DOI: 10.5281/zenodo.21305493). The sequencing data generated in this study have been submitted to the NCBI database under BioProject ID PRJNA 1497264 and are currently being processed according to NCBI’s requirements for large targeted-locus submissions.

## Supplementary Materials

The following supporting information can be downloaded at: https://www.mdpi.com/article/10.3390/insects17070746/s1, Figure S1. A Total amino acid content in *Clanis bilineata larvae*. B Total fatty acid content in *Clanis bilineata larvae*. The asterisk indicates significant difference (* *p* < 0.05; ** *p* < 0.01; *** *p* < 0.001; independent samples *t*-test). Figure S2. Microbial composition in each biological replicate of soil, leaves, and CBTL. A Relative abundance of soil microorganisms at the genus level across replicates in the CBTL treatment; B Relative abundance of leaf microorganisms at the genus level across replicates.

## Abbreviations

The following abbreviations are used in this manuscript:

AA	Amino acid
AK	Available potassium
Ala	Alanine
Arg	Arginine
AN	Available nitrogen
AP	Available phosphorus
CBTL	*Clanis bilineata tsingtauica* larvae.
COG	Clusters of orthologous groups
C16: 0	Palmitic acid
C18: 0	Stearic acid
ET	Ethylene
FA	Fatty acid
Gly	Glycine
GOT	Glutamate oxaloacetate transaminase
GPT	Glutamate pyruvate transaminase
GS	Glutamine synthetase
HA	Huaian
JA	Jasmonic acid
LYG	Lianyungang
NJ	Nanjing
PCoA	Principal coordinate analysis
Ser	Serine
SOM	Soil organic matter
TK	Total potassium
TN	Total nitrogen
TP	Total phosphorus
t-C18: 1	Elaidic acid
t,t-C18: 2	Trans-Linoleic acid
Tyr	Tyrosine
