# Supplementary material for: Regional Soil and Soybean-Associated Bacterial Communities Are Linked to Larval Nutritional Composition in the Edible Insect Clanis bilineata tsingtauica
Source: Insects. 2026 Jul 21;17(7):746. doi: 10.3390/insects17070746 (PMC13410249; doi:10.3390/insects17070746)
Supplement: Supplementary file 1 [file insects-17-00746-s001.zip › insects-4398950-supplementary.pdf]

Regional soil and soybean–associated bacterial communities are linked to larval nutritional composition in the edible insect *Clanis bilineata tsingtauica*

Zong-Nan Li [1,†], Qing-Yi Li [1,2,†], Qiu-Lin Wu [3], Su-Fen Cui [4], Pan Deng [1], Ji-Hu Li [5], Jian-Jun Guo [6], Lei Qian [1,*], and Huai-Jian Liao [1,2,*]

[1] *Institute of Leisure Agriculture, Jiangsu Academy of Agricultural Sciences, Nanjing 210014, China*

[2] *College of Biotechnology, Jiangsu University of Science and Technology, Zhenjiang 212100, China*

[3] *Jiangsu Provincial University Key laboratory of Agricultural and Ecological Meteorology; School of Ecology and Applied Meteorology, Nanjing University of Information Science and Technology, Nanjing 210044, China*

[4] *School of Grain Science and Technology, Jiangsu University of Science and Technology, Zhenjiang 212004, China*

[5] *Institute of Nanfan and Seed Industry, Guangdong Academy of Sciences, Guangzhou 510000, China*

[6] *Institute of Entomology, Guizhou University, Guiyang 550025, China*

* *Correspondence: qianlei14930@sina.cn (Qian L.); lhj@jaas.ac.cn (Liao H.)*

† *These authors contributed equally to this work*

Comparison of total amino acid and total fatty acid content

Analysis of total amino acid and total fatty acid contents in *C. bilineata tsingtauica* larvae from the three regions, based on individual amino acid and fatty acid levels, revealed that the total amino acid content in LYG larvae was significantly higher than in HA larvae ($P < 0.05$), whereas the total fatty acid content in HA larvae was significantly higher than in both LYG and NJ larvae ($P < 0.05$). These findings are consistent with the trends observed for individual amino acids and fatty acids. Compared with individual fatty acids, the differences in total fatty acid content among the three regions were more pronounced. The cumulative effect of multiple fatty acids intensified the distinction between HA and NJ larvae, reaching statistical significance. The observed differences in total amino acids and total fatty acids among the three regions further illustrate the influence of geographical variation on the nutritional profile of *C. bilineata tsingtauica* larvae. Additionally, the inverse relationship between amino acid and fatty acid levels provides evidence of the impact of soybean saponins on larval nutritional metabolism.

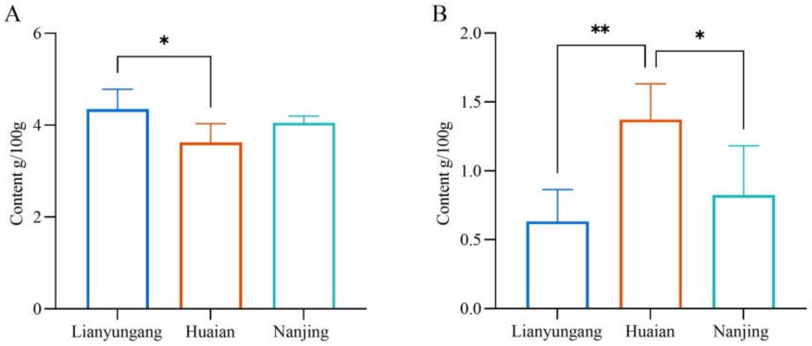

Fig. S1. A Total amino acid content in *C. bilineata tsingtauica* larvae. B Total fatty acid content in *C. bilineata tsingtauica* larvae. The asterisk indicates significant difference (*$P < 0.05$; **$P < 0.01$; ***$P < 0.001$; independent samples t-test).

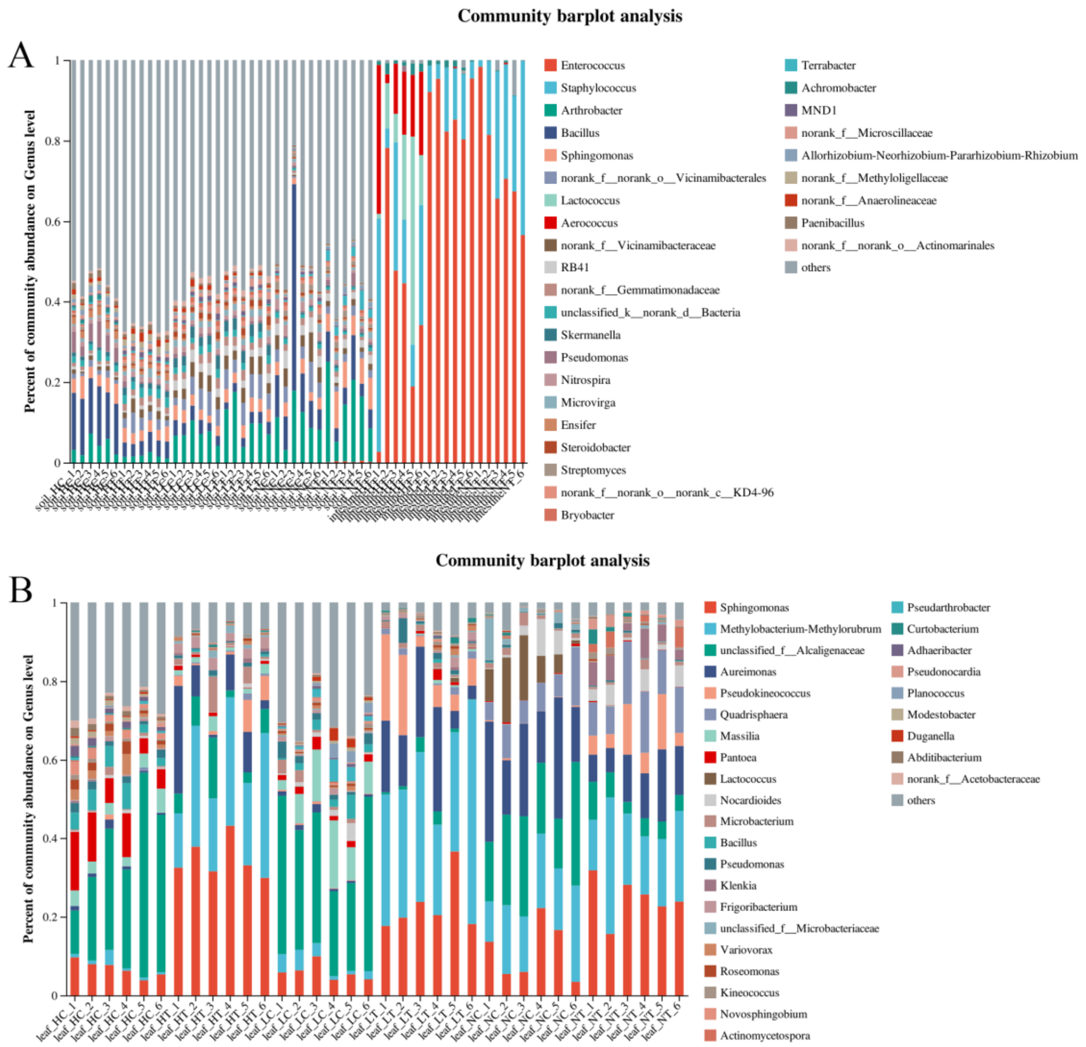

Fig. S2. Microbial composition in each biological replicate of soil, leaves, and CBTL. A Relative abundance of soil microorganisms at the genus level across replicates in the CBTL treatment; B Relative abundance of leaf microorganisms at the genus level across replicates.
